# Evaluation of nationwide supplementary immunization in Lao People's Democratic Republic: Population-based seroprevalence survey of anti-measles and anti-rubella IgG in children and adults, mathematical modelling and a stability testing of the vaccine

**DOI:** 10.1371/journal.pone.0194931

**Published:** 2018-03-29

**Authors:** Masahiko Hachiya, Shinsuke Miyano, Yoshio Mori, Emilia Vynnycky, Phath Keungsaneth, Phengta Vongphrachanh, Anonh Xeuatvongsa, Thongchanh Sisouk, Vilasak Som-Oulay, Bouaphan Khamphaphongphane, Bounthanom Sengkeopaseuth, Chansay Pathammavong, Kongxay Phounphenghak, Tomomi Kitamura, Makoto Takeda, Katsuhiro Komase

**Affiliations:** 1 Bureau of International Health Cooperation, National Center for Global Health and Medicine, Shinjuku, Tokyo, Japan; 2 Department of Virology 3, National Institute of Infectious Diseases, Musashimurayama, Tokyo, Japan; 3 Modelling and Economics Unit, Public Health England, London, United Kingdom; 4 TB Modelling Group and TB Centre, London School of Hygiene & Tropical Medicine, London, United Kingdom; 5 Centre for Mathematical Modelling of Infectious Diseases, London School of Hygiene & Tropical Medicine, London, United Kingdom; 6 Department of Hygiene and Health Promotion, Ministry of Health, Vientiane Capital, Lao PDR; 7 National Center for Laboratory and Epidemiology, Ministry of Health, Vientiane Capital, Lao PDR; 8 National Immunization Program, Ministry of Health, Vientiane Capital, Lao PDR; Public Health England, UNITED KINGDOM

## Abstract

**Background:**

Measles outbreaks have occurred in some countries despite supplementary immunization activities (SIA) using measles-containing vaccine with high vaccination coverage. We conducted a cross-sectional seroprevalence survey to estimate population immunity in Lao People's Democratic Republic where repeated mass immunization has failed to eliminate measles.

**Methods and findings:**

In this nationwide multistage cluster sampling survey conducted in 2014 based on probability proportionate to size sampling, blood samples were collected from 2,135 children and adults living in 52 randomly selected villages. Anti-measles and anti-rubella IgG were measured, and IgG prevalence was calculated. We applied mathematical modelling to estimate the number of cases of congenital rubella syndrome (CRS) in 2013 that were averted by the 2011 SIA. A stability testing was applied to the MR vaccine at 4°C, 25°C, and 35°C to examine stability differences between measles and rubella vaccine components. Measles IgG prevalence was significantly lower in the target age groups (5–21 years) of the 2011 SIA using a combination vaccine for measles and rubella vaccine (MR vaccine) than in young adults (22–39 years) (86.8% [95% CI: 83.0–90.6] vs. 99.0% [98.3–99.8]; p<0.001), whereas rubella IgG prevalence was significantly higher (88.2% [84.5–91.8] vs. 74.6% [70.7–78.5]; p<0.001). In the SIA target age groups, prevalence of measles IgG, but not rubella IgG, increased with age. CRS cases prevented in 2013 ranged from 16 [0–50] to 92 [32–180] if the force of infection had remained unchanged or had been reduced by 75%, respectively. In freeze-dried conditions, the measles vaccine component was more heat sensitive than the rubella component.

**Conclusions:**

Inconsistent IgG prevalence between measles and rubella in Lao PDR can be partly explained by different stability of the measles and rubella vaccine components under heat exposure. Suboptimal vaccine handling may cause insufficient immunogenicity for measles, which subsequently leads to an outbreak despite high SIA coverage, while direct evidence is lacking. Temperature monitoring of the vaccine should be conducted.

## Introduction

Measles and rubella are vaccine-preventable viral diseases that remain important causes of death and disability, especially in countries with limited health systems. Measles killed 2.6 million people globally every year before the measles vaccine came into widespread use, and still killed 145,700 infants before their first birthday in 2013 despite 83–84% measles immunization coverage for the infant population worldwide [1–3]. Rubella is a mild self-limited illness occurring mainly in children, but infection before conception or during early pregnancy may cause miscarriage, foetal death, or severe congenital defects known as congenital rubella syndrome (CRS) [4]. More than 100,000 babies are estimated to be born with CRS worldwide annually [4, 5, 6, 7].

Lao People’s Democratic Republic (PDR) is a landlocked developing country with poor infrastructure, with 10% to 13% of vaccine procurement funded under the national budget. Measles is one of the country’s priority vaccine-preventable diseases and an expanded programme on immunization (EPI) started in 1984. Under this EPI, measles-containing vaccination coverage among 12 month olds increased from 6% to 42% between 1984 and 2000 [Ministry of Health]. After several outbreaks occurred, nationwide SIA were implemented in 2001 (86% coverage), 2007 (96% coverage), and 2011 (97% coverage) using measles-containing vaccines. A monovalent vaccine was used both for the 2001 SIA, targeting children aged 9 months to 4 years, and the 2007 SIA, targeting children aged 9 months to 14 years, and the combination vaccine for measles and rubella (MR vaccine) was introduced for the 2011 SIA targeting children aged 9 months to 19 years. Despite these efforts, the country continued to experience measles outbreaks. For example, even as measles immunization coverage increased from 50% to 82% from 2001 to 2013 and SIA coverage was 96% and 97% in 2007 and 2011, respectively, measles outbreaks occurred in 2012, 2013, and 2014 [Ministry of Health]. The exact causes of the measles outbreaks are unknown and need to be investigated to prevent further outbreaks.

To this end, we conducted a nationwide multistage random cluster sampling survey in 2014 to measure anti-measles and anti-rubella IgG prevalence among children and adults in Lao PDR with the aims of estimating population immunity, evaluating previous vaccination effectiveness, and then estimating through mathematical modelling the number of CRS cases averted by the 2011 SIA. We also conducted a stability test to evaluate the stability of measles and rubella vaccine components, since the information may be useful to understand differences between rubella and measles seroprevalence.

## Materials and methods

We conducted a cross-sectional seroprevalence survey of anti-measles IgG and anti-rubella IgG in children and adults in 2014 using representative samples from all 143 districts of Lao PDR using a multistage random cluster sampling design.

### Study population

The population covered by the 2011 SIA was aged 3–21 years at the time of the survey. Because we aimed to evaluate the 2011 SIA, we included both the populations who would have definitely been covered and not covered by the 2011 SIA, namely those aged 5–21 years and 1–2 and >21 year olds respectively and compared the seroprevalence between them. Those aged 3–4 years were not included, because their immunization history and date of birth were difficult to determine as calendar and traditional ages are often confused in rural villages [8, 9] (Table 1).

**Table 1:** Reported coverage of routine immunization and supplementary immunization activities with measles containing vaccine by age at the survey in Lao PDR, 2014*.

Age at the survey (years)	Year of birth	Routine MCV coverage (%)	2001 SIA using MCV	2007 SIA using MCV	2011 SIA using MRCV
1	2012	72			
2	2011	69			
3	2010	64			〇
4	2009	59			〇
5	2008	52			〇
6	2007	40			〇
7	2006	48		〇	〇
8	2005	41		〇	〇
9	2004	36		〇	〇
10	2003	42		〇	〇
11	2002	55		〇	〇
12	2001	50		〇	〇
13	2000	42	〇	〇	〇
14	1999	71	〇	〇	〇
15	1998	71	〇	〇	〇
16	1997	67	〇	〇	〇
17	1996	73		〇	〇
18	1995	68		〇	〇
19	1994	73		〇	〇
20	1993	46		〇	〇
21	1992	46			〇
22	1991	47			
23	1990	32			
24	1989	20			
25	1988	23			
26	1987	11			
27	1986	10			
28	1985	6			
29	1984	6			
30	1983	7			

*Reported coverage of SIAs in 2001, 2007, and 2011 are 86%, 96%, and 97%, respectively

SIA, supplementary immunization activities; MCV, measles containing vaccine; MRCV, measles and rubella containing vaccine

### Sample size calculation

The required sample size (n) was calculated using the formula

$$n=Z^{2}\times p(1-p)DEFF/\;(d^{2}\times RR)=$$

where *n* = sample size

*Z* = significance level for 95% confidence

*p* = expected prevalence

*DEFF* = design effect

*d* = precision

*RR* = response rate

In this, we used a 5% level of significance (Z = 1.96, the z-score needed for this in a two-tailed test)), precision (d) of ±0.05 to ±0.06, and expected measles and rubella IgG-positive rates (p) of 60% for children aged 1–2 years and 90% for children aged > 5 years and adults, an expected design effect (DEFF) of 1.6, and a response rate (RR) of 99%. The required sample size was calculated to be 416 samples for 1–2 year olds and 312 samples each for 5–14 year olds, 15–19 year olds, and adults aged 40 years old or more. We previously found that adults working in rice fields can be difficult to sample [8, 9], so we included 832 parents of children aged 1–2 years. The survey required collection of 2,184 samples in total.

### Survey design and sampling

A three-stage random cluster sampling design was adopted, as probability sampling is recommended by the World Health Organization [10]. For the first stage, 26 of 143 districts were randomly selected by applying probability proportionate to size (PPS) sampling based on the latest population census of 2005 obtained from the Department of Statistics, Lao PDR. For the second stage, 2 villages were randomly selected from each district by PPS sampling. For the third stage, 42 participants were randomly selected using a paper-based lottery from a list of households in each village satisfying the required sample size: 8 aged 1–2 years, 6 aged 5–14 years, 6 aged 15–19 years, 16 parents of the 1–2 year olds, and 6 aged ≥ 40 years. We chose these age groups for better field practicability because we used several registers as sources of the resident lists (i.e., EPI register, poverty reduction programme log, and village head’s register).

### Data collection

The survey was conducted for 2 weeks from 27 January to 7 February, 2014. A survey team (2 members per team) was organized in each of the 26 selected districts and was expected to investigate individuals sampled from 2 villages in the district. For smooth implementation of the survey, team members were recruited from among district health officers in the district because of their familiarity with the local cultural context and security information. The teams had two tasks for the investigation: (i) a brief face-to-face interview to obtain demographic information and (ii) blood collection using a dried blood spot method by finger prick [11]. To ensure compliance with survey procedures, team members were trained and supervised by national government staff from the National Immunization Programme, National Centre for Laboratory and Epidemiology, and the Ministry of Health as well as provincial health officers, Lao PDR.

### Anti-measles and anti-rubella IgG titres

A small amount of blood was spotted onto Whatman 903 Protein Saver filter paper (Whatman, Maidstone, Kent, UK) by finger prick and air-dried for at least 60 min [11]. The filter papers were sealed in plastic bags and transported to Japan within a few weeks. Blood samples were extracted from dried blood spots on the filter paper [12], and IgG levels were measured using commercially available enzyme-linked immunosorbent assay (ELISA) kits (Enzygnost Anti-Measles Virus/IgG and Anti-Rubella Virus/IgG, Siemens Healthcare Diagnostics) according to the manufacturer’s instructions at the Department of Virology 3, National Institute of Infectious Diseases, Japan. Optical density values were converted to quantitative data, and the results were considered positive at higher than 120 mIU/mL for measles and 10 IU/mL for rubella [13, 14].

### Estimation of the number of averted CRS cases, by mathematical modelling

Adapting methods used previously [7, 15–17], we first fitted four age-structured catalytic models to the observed weighted age-stratified serological data for those aged >21 years using maximum likelihood to estimate the average force of infection (the rate at which susceptibles are infected) that children (< 15 years) and adults (≥ 15 years) experienced before the 2011 SIA was introduced. The fitting just used data for those aged > 21 years in 2014 because they were outside the age groups targeted by the 2011 SIA and so their seroprevalence would not have been directly affected by vaccination. The force of infection was assumed to differ (models A and B) or be identical (models C and D) for the ages <15 and ≥15 years. The sensitivity of the rubella serological (antibody) assay was either estimated (models A and C) or assumed to be 100% (models B and D). The models assumed that maternal immunity lasts for 6 months and we explored the effect of assuming that the force of infection after the SIA was reduced by either 0%, 25%, 50%, 75%, or 100%). The Supplementary Information provides further details about the models and the equations. The force of infection used in subsequent calculations (see below) was the one obtained from the model selected according to biological plausibility, as described elsewhere [17].

The best-fitting value for the force of infection from the selected model was used to estimate the CRS incidence per 100,000 live births in 2013 among women in 5 year age groups between 15 and 44 years and to estimate the CRS incidence if the SIA had not taken place in 2011. The CRS incidence per 100,000 live births among women in age group A was given by the following expression:

$$0.65\times s_{u}(A)(1-e^{-16\lambda_{o}(1-r)/52})\times 100000$$

where *s*_*u*_*(A)* is the proportion of women in age group A that are susceptible, *λ*_*0*_ is the average force of infection among those aged ≥ 15 years before the SIA was introduced, and *r* is the average reduction in the force of infection after the SIA was introduced. For participants aged 15–21 years, *s*_*u*_*(A)* was taken to equal the observed proportion of women in age group *A* who were seronegative during the seroprevalence survey. For the other age groups, s_U_(A) was calculated using the appropriate expressions from the model (see Supplement). As in previous analyses [14], the risk of a child being born with CRS was assumed to be 65% when the mother was infected during the first 16 weeks of pregnancy and zero thereafter. The CRS incidence per 100,000 live births among women aged 15–44 years was calculated using the same approach used elsewhere [14], as the mean CRS incidence per 100,000 live births for each 5-year maternal age group, weighted by the number of live births occurring for each maternal age group in 2013. To obtain the number of infants with CRS born to women in each age group, the CRS incidence per live birth among women in each age group for Lao PDR was multiplied by the estimated number of live births among women in corresponding age groups in 2013 for Lao PDR. These were then summed over the ages of 15–44 years to give the total number of infants born with CRS in 2013. The number of live births among women in a given age group was calculated by multiplying the age-specific fertility rates by the number of females in each age group in 2013, both of which were extracted from the UN population database, 2012 revision. The number of CRS cases in 2013 prevented by the 2011 SIA was calculated as the difference between the total number of CRS cases calculated using the CRS incidence with the SIA in place and that calculated if the SIA had not been implemented.

Confidence intervals (CIs) for the force of infection and CRS incidence for each catalytic model, and the number of CRS cases prevented by the SIA, were obtained by bootstrapping using 1,000 bootstrap datasets generated with the approach employed by Shkedy et al. [18].

### Stability testing of MR vaccines

To evaluate differences in heat sensitivity between two viruses (Edmonston-Zagreb measles virus and Wistar RA27/3 rubella virus), a single lot of the MR vaccine (MR-VAC, Serum Institute of India) underwent a stability testing under both freeze-dried and reconstituted conditions. Freeze-dried vaccines were incubated at 4°C, 25°C, or 35°C for 1, 3, 7, 14, 21, or 28 days and stored at –80°C until used for titration. Reconstituted vaccines were incubated at 4°C, 25°C, or 35°C for 1, 3, 6, 12, or 24 h or 2, 4, or 7 days and stored at –80°C until used for titration.

Infectivity titres of the measles vaccine component in the incubated MR vaccine was determined by the standard plaque assay on African green monkey kidney-derived Vero cells. Since the rubella vaccine component also produces plaques on Vero cells, the rubella vaccine component was neutralized by the anti-rubella virus antibody for 1 h at 4°C before incubation of Vero cells with the vaccine samples.

Infectivity titres of the rubella vaccine component in the incubated MR vaccine was determined by the standard plaque assay on the rabbit kidney-derived RK-13 cells [19]. The measles vaccine component was not neutralized by the anti-measles virus antibody before titration, because the measles vaccine component did not produce plaque on RK-13 cells and the plaque numbers of rubella vaccine component were not affected by the neutralization of the measles vaccine component (data not shown).

### Data entry and statistical analysis

All of the collected data were double-entered and cleaned on a Microsoft Excel 2013 spreadsheet. The statistical analysis was conducted using STATA version 13 and 14 (Stata Corp., College Station, TX, USA). Calculations of the IgG prevalence among the participants considered the multistage cluster sampling design and sampling weight of each participant to elicit representative, unbiased results. Differences between the immunity of each age group were examined with Chi-square and Fisher’s exact tests. Statistical significance was set at p<0.05.

### Ethical considerations

The surveyors explained the detailed survey objectives and procedures to the local authorities and selected participants verbally and in writing. Written informed consent was obtained from all selected participants. When participants were aged < 15 years, they were explained the survey objectives and procedures according to their level of understanding, and consent was obtained from their parents or legal guardians. Participants’ names were not recorded. The research proposal was approved by the National Center for Global Health and Medicine (Japan, NCGM-G-001459-00), the ethics committee of the Ministry of Health (Lao PDR, 025-NECHR), and the National Institute of Infectious Diseases (Japan, NIID-494).

## Results

The survey teams visited all 52 selected villages from the 26 districts and completed blood sampling in 2,153 subjects. After 18 subjects were excluded from the study due to missing data, this left data for 2,135 subjects (97.8% of the required sample size) for analysis. Mean age was 23.2 years, ranging from 1 to 81 years (95% CI: 22.5–23.9). Males accounted for 44.8% of all selected subjects. The places of residence were the north for 26.9%, central region for 26.9%, and south for 46.3%. Regarding ethnicity, 70.3% were Laolum and 29.7% were other minorities. The average of the reported routine immunization coverage for measles containing vaccine was 71.0% [95% CI: 55.5–86.5]) in selected districts in 2012.

### Estimated IgG prevalence of measles and rubella

Of the 2,135 selected subjects, 1,733 and 1,469 tested positive for measles and rubella IgG, respectively. Estimated IgG prevalence was 83.9% for measles (95% CI: 83.8–84.0) and 75.4% for rubella (95% CI: 75.3–75.5) after considering the sampling design and individual sampling weight.

**Fig 1:**
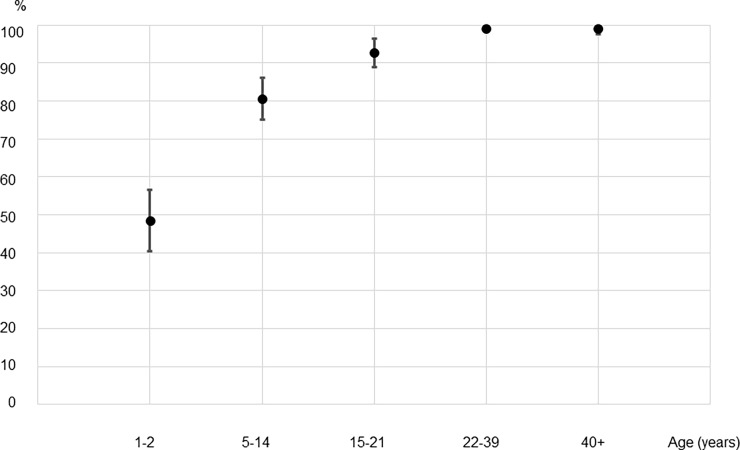
Measles IgG seroprevalence measured by ELISA in different age groups from representative populations of Lao PDR, 2014. *An ELISA value of 120 mIU/mL was considered positive. †Individuals 5–14 years old and 15–21 years old were among the targeted age groups of supplementary immunization activities conducted in 2011 using the MR combination vaccine with coverage of 97%. Their IgG prevalence was estimated to be 86.8% (95% CI: 83.0–90.6).

**Fig 2:**
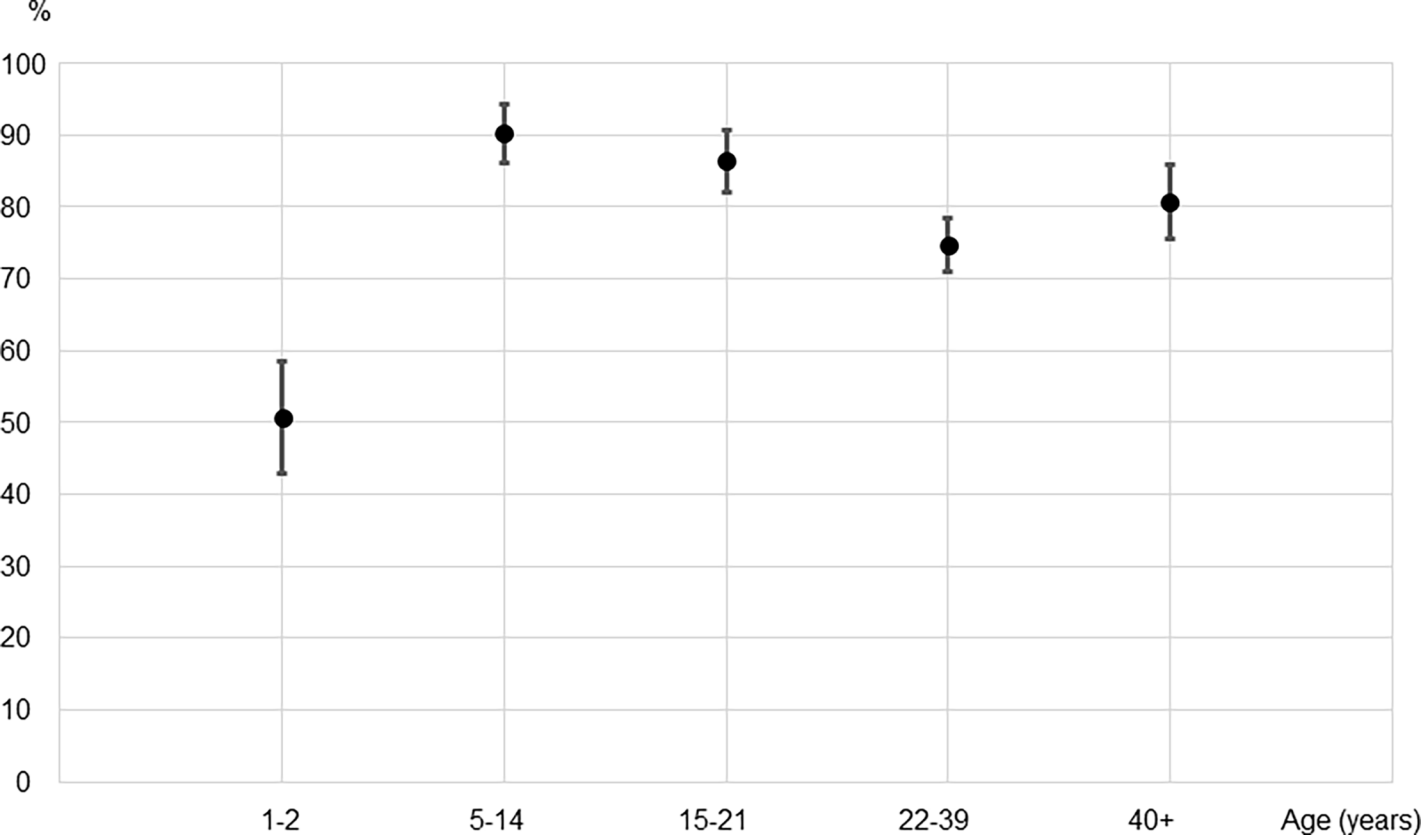
Rubella IgG seroprevalence measured by ELISA in different age groups from representative populations in Lao PDR, 2014. *An ELISA value of 10 IU/ml was considered positive. †Individuals 5 to 14 years old and 15 to 21 years old were among the targeted age groups of supplementary immunization activities conducted in 2011 using the MR combination vaccine with coverage of 97%. Their IgG prevalence was estimated to be 88.2% (95%CI: 84.5–91.8).

As shown in Fig 1, the seroprevalence for measles IgG for 5–21 year olds, who would have been vaccinated during the 2011 SIA was significantly higher than that of 1–2 year olds who were born since the SIA (86.8% [95% CI: 83.0–90.6] vs. 48.6% [95% CI: 40.0–57.1]; p<0.001), but lower than that of 22–39 year olds (86.8% [95% CI: 83.0–90.6] vs. 99.0% [95% CI: 98.3–99.8]; p<0.001) who were not targeted by the SIA. In contrast (Fig 2), the seroprevalence for rubella IgG for 5–21 year olds was significantly higher than that for people who were not vaccinated during the SIA (88.2% [95% CI: 84.5–91.8] vs. 50.7% [95% CI: 42.5–58.9] and 74.6% [95% CI: 70.7–78.5] for 1–2 year olds and 22–39 year olds respectively, p<0.001 for both).

To examine whether immunity differed among the SIA-targeted age groups, we compared measles and rubella IgG positivity between 5–14 and 15–21 year olds. The IgG-positive rate was significantly different for measles—80.6% [95%CI: 74.8–86.3] and 92.7% [95%CI: 88.8–96.6], respectively (p<0.001)—but not for rubella—90.2% [95%CI: 85.8–94.5] and 86.3% [95%CI: (81.7–90.9], respectively.

### Model-based estimates of CRS cases averted

**Fig 3:**
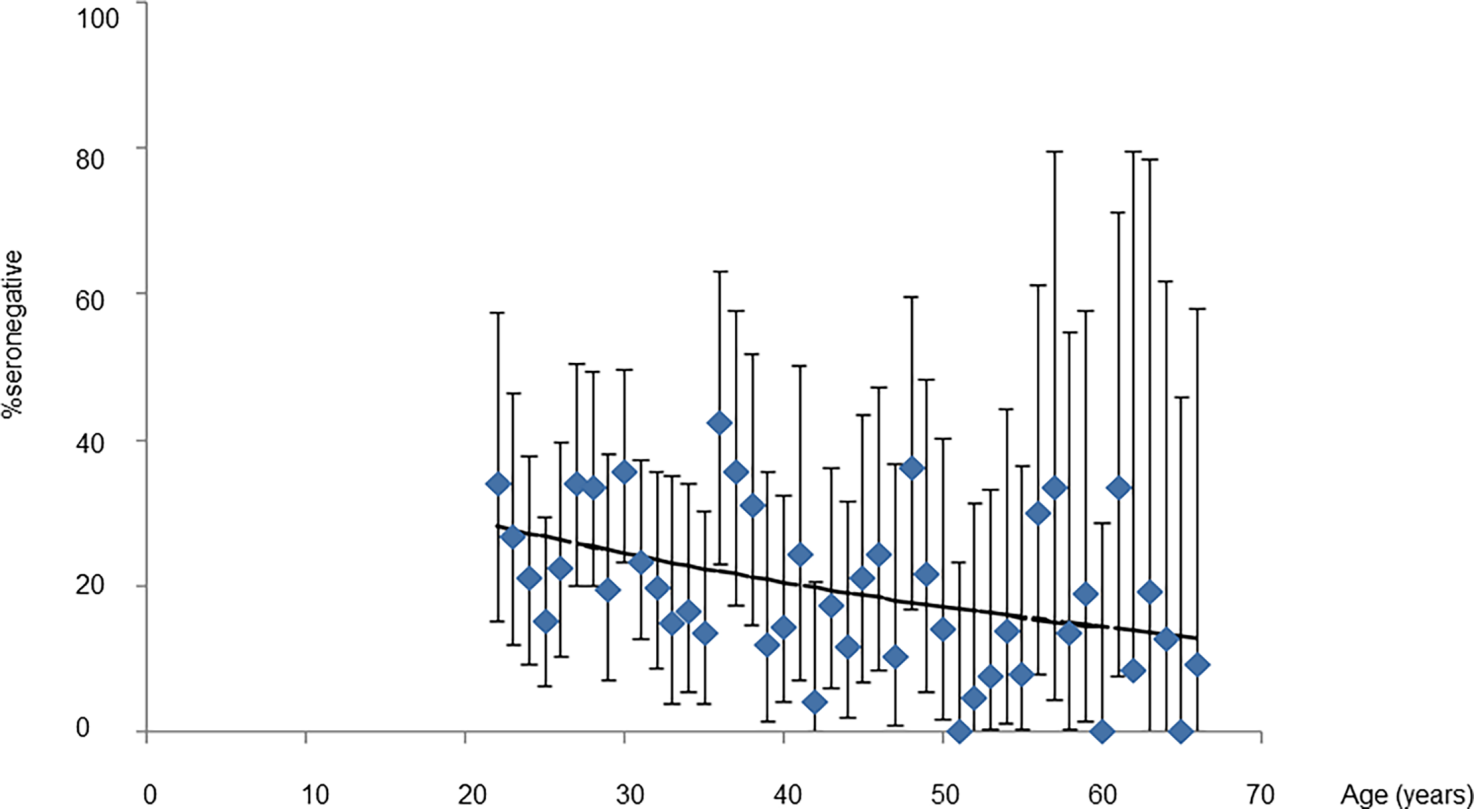
Comparison between the predictions of age-specific percentage seronegative, obtained using the best-fitting catalytic model and the observed data. Bars reflect 95% confidence intervals of the observed data. Lines reflect predictions from the best-fitting model.

Fig 3 compares the best-fitting predictions from the selected catalytic model (B) of the age-specific percentage seronegative against the observed data. For reference, S1 Table in the supplement summarises the estimates obtained from each of the catalytic models. Table 1 summarizes the best-fitting estimates of the average force of infection before the 2011 SIA, the CRS incidence in 2013 and the number of CRS cases in 2013 prevented by the 2011 SIA for different assumptions about the reduction in the force of infection after the SIA. For all assumed values for the reduction in the force of infection after the 2011 SIA (Fig 3), the fit of the model to the data was similar, as were the best-fitting values for the force of infection, although the latter had wide CIs (Table 2). Assuming that the force of infection decreased by 50% after the SIA, the force of infection was estimated to be 80 (95% CI: 63–97) and 18 per 1000 (95% CI: 8–30) per 1000 susceptibles for those aged <15 years and ≥ 15 years respectively.

**Table 2:** Best-fitting values of the force of infection in Lao PDR before the 2011 SIA among participants aged < 15 and ≥ 15 years, obtained by the selected best-fitting model (model B) for different assumptions about the reduction in the force of infection after the SIA, and CRS incidence per 100,000 live births among women aged 15–44 years after weighting by the number of live births occurring among women in different maternal age groups.

Assumed reduction in force of infection since 2011	Force of infection per 1000 (susceptibles) before 2011		Loglike-lihood deviance (degrees of freedom)	Weighted CRS incidence per 100,000 live births		Number of CRS cases in 2013		
	<15 years old	≥15 years old		Without vaccination	With vaccination	Without vaccination	With vaccination	Prevented
0%	79 (61,96)	18 (8,29)	61 (43)	95 (37,178)	85 (35,152)	158 (62,297)	142 (58,254)	16 (0,50)
25%	79 (62,97)	18 (8,29)	61 (43)	94 (37,175)	69 (28,122)	158 (62,293)	116 (47,204)	42 (11,92)
50%	80 (63,97)	18 (8,30)	61 (43)	93 (37,172)	53 (22,94)	156 (61,288)	89 (36,156)	67 (21,136)
75%	80 (64,97)	18 (8,29)	61 (43)	92 (36,170)	37 (15,66)	154 (61,285)	62 (26,111)	92 (32,180)
100%	81 (65,98)	18 (8,29)	61 (43)	92 (36,168)	21 (8,38)	153 (61,280)	35 (14,64)	118 (43,224)

Note. Confidence intervals were obtained by bootstrapping. Columns labelled “without vaccination” refer to estimated CRS incidence that might have occurred in 2013 if the SIA had not been implemented.

If the SIA had not been implemented in 2011, the CRS incidence was estimated to have been approximately 93 per 100,000 live births (93 [95% CI: 37–172]) if the SIA was assumed to have reduced the force of infection by 50% (Table 2). This corresponds to almost 160 infants being born with CRS in 2013 (156 [95% CI: 61–288]).

The estimated CRS incidence after the SIA was sensitive to the assumed reduction in the force of infection following the 2011 SIA (Table 2), ranging from 85 per 100,000 live births [95%CI: 35–152] assuming no reduction in the force of infection to 37 per 100,000 live births (95% CI: 15–66) assuming a 75% reduction. The corresponding estimated numbers of CRS cases were 142[95%CI: 58–254] and 62 [95%CI: 26–111] babies born with CRS in 2013, respectively (Table 2). The number of CRS cases prevented in 2013 ranged from 16 [95%CI: 0–50] assuming no reduction in the force of infection to 92 [95%CI: 32–180] assuming a 75% reduction after the SIA had been introduced.

### Stability test results

**Fig 4:**
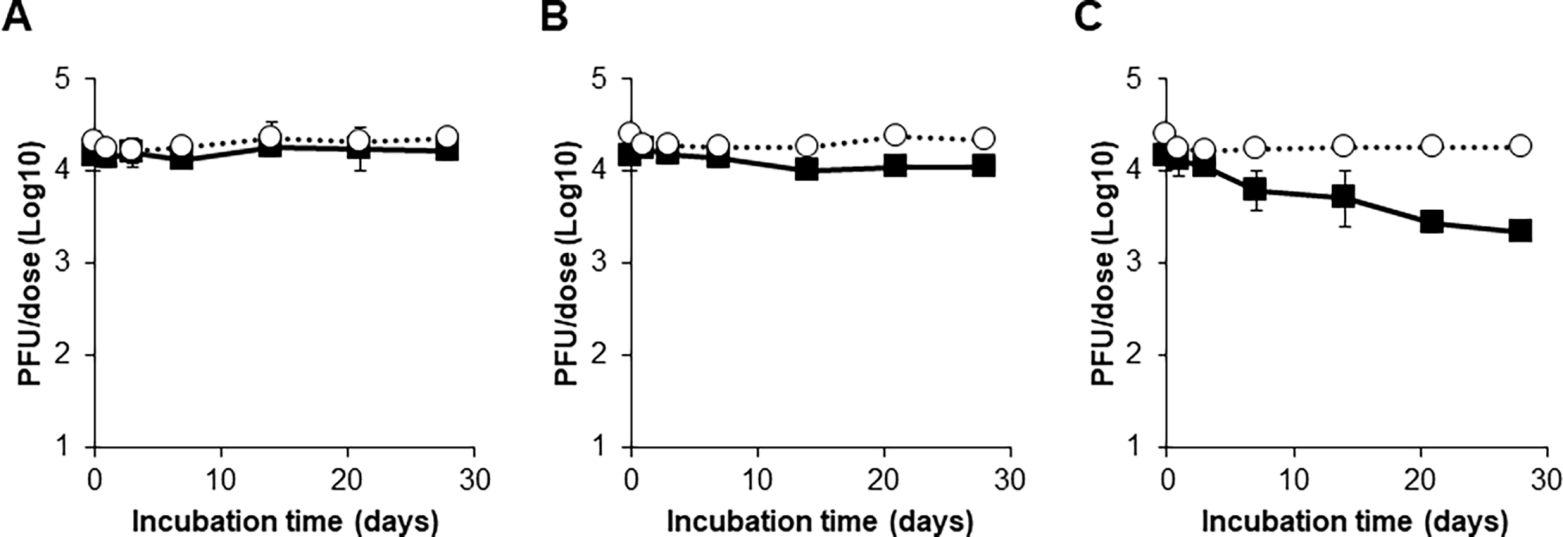
Stability testing of freeze-dried measles and rubella combination vaccines. After incubation of freeze-dried vaccines at 4°C (A), 25°C (B), or 35°C (C), titres of measles and rubella viruses in the vaccines were measured by plaque assays. Filled squares and open circles indicate titres of measles and rubella viruses, respectively. Means of three vials are shown. Error bars indicate standard deviations.

The infectious titre of the measles vaccine component was stable and remained over 10^4^ PFU/dose after incubation of the freeze-dried vaccine at 4°C or 25°C for 28 days (Fig 4). The infectious titre of the measles vaccine component decreased day by day at 35°C incubation. Although the titre was still higher than 10^3^ PFU/dose, which is the minimum potency requirement of the measles vaccine, an approximately 85% reduction in the titre was observed after incubation for 28 days. In contrast, incubation at all 3 different temperatures until 28 days did not affect the infectious titre of the rubella vaccine component in the freeze-dried vaccines.

**Fig 5:**
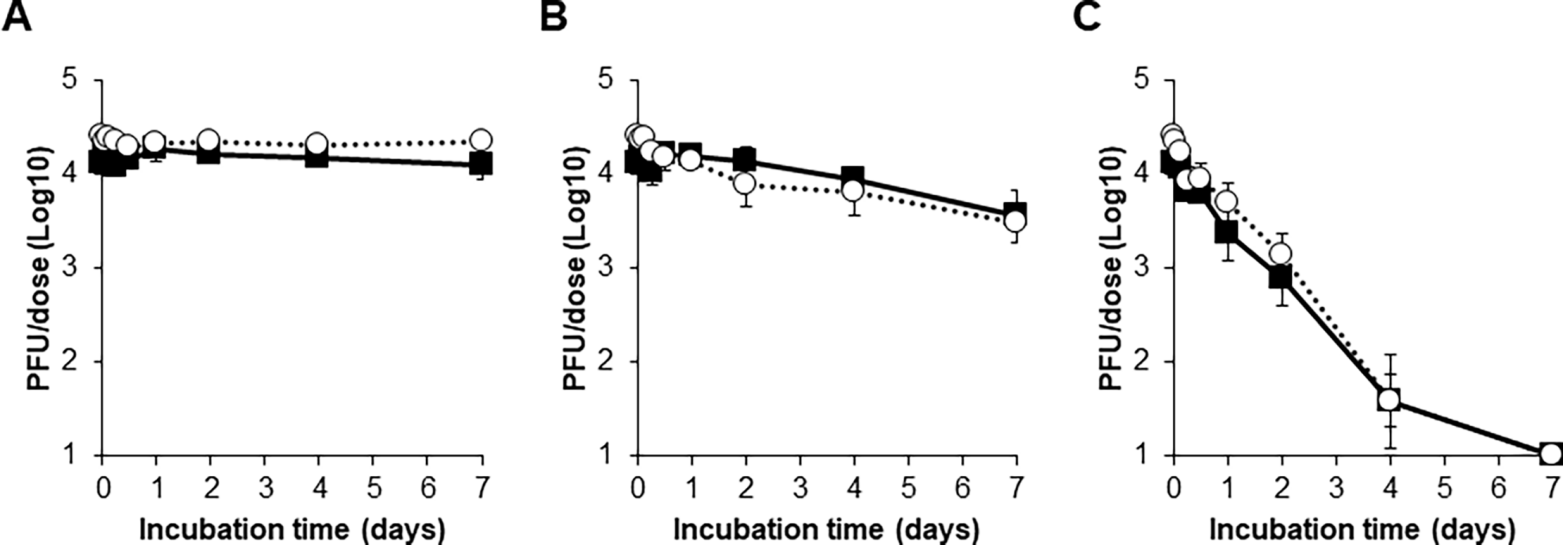
Stability testing of reconstituted measles and rubella combination vaccines. After incubation of reconstituted vaccines at 4°C (A), 25°C (B), or 35°C (C), titres of measles and rubella viruses in the vaccines were measured by plaque assays. Filled squares and open circles indicate titres of measles and rubella viruses, respectively. Means of three vials are shown. Error bars indicate standard deviations.

When vaccines were reconstituted and then incubated, the infectious titres of both vaccine components were stable during incubation at 4°C, whereas they reduced day by day during incubation at 25°C or 35°C (Fig 5). In particular, by incubation at 35°C, the infectious titres of the measles and rubella vaccine components became less than 10^3^ PFU/dose by 2 and 4 days, respectively, and were less than 10 PFU/dose by 7 days. However, there were no clear differences between the stability kinetics of the measles and rubella vaccine components at all temperatures.

## Discussion

Our study has three major findings. First, the measles IgG prevalence estimated from ELISA results for the 2011 SIA-targeted age groups was lower than the reported immunization coverage, and it was lower than the measles IgG prevalence estimated for young adults. Second, in contrast, the estimated rubella IgG prevalence for the 2011 SIA-targeted age groups was higher than that for young adults, who were unlikely to be vaccinated, and the estimated number of CRS cases averted by the 2011 SIA ranged from 16 to 92 cases in 2013, according to mathematical modelling. Third, the measles component in the same vaccine used during the 2011 SIA was more heat sensitive than the rubella component under freeze-dried conditions.

### Population immunity among the target age groups of the 2011 SIA

The anti-rubella IgG prevalence measured by IgG ELISA in the SIA target age groups (88.2%) reached and even exceeded the immunization coverage threshold of 80% recommended by WHO to avoid the potential for an increase in the CRS burden following the introduction of rubella-containing vaccination [6]. This high IgG seroprevalence would have been obtained by the 2011 SIA, when rubella vaccine was introduced in the country for the first time, because the seroprevalence in our study among those targeted by the SIA was higher than that of people aged 22–39 years (88.2% and 74.6% respectively; p<0.001), who would have acquired rubella immunity only by natural infection.

On the other hand, anti-measles IgG prevalence measured by ELISA in the SIA target age groups did not reach the required threshold for effective measles control, which is generally considered to be 93%-94% based on its basic reproduction number (R_0_) of 12–18 [2]. Young adults aged 22 to 39 years likely acquired immunity against measles mainly by natural infection, as routine measles elimination effort did not start until 2000. Despite a high reported coverage for the 2007 and 2011 SIAs (96% and 97% respectively), the comparatively low seroprevalence seen in our study among those targeted (7–20 and 5–21 year olds in our survey) suggest that they did not adequately raise population immunity. Moreover, the increase in the measles IgG with age in the SIA target groups (80.6% in 5–14 years and 92.7% in 15–21 years, p<0.001) implies that wild measles virus is still circulating in the community.

### Model-based estimates of the number of CRS cases averted

We estimated that the number of CRS cases averted by the 2011 SIA ranged from 16 (95% CI: 0–50) to 92 (32–180) in 2013. The most important and serious consequence of rubella is CRS. The risk of CRS depends on the gestational age at the time of maternal infection [20]. Rubella vaccinations play a major role in preventing infants being born with CRS. Although many countries have recently newly introduced rubella vaccine through SIA and routine immunization, few have reported the impact of vaccination [21–23]. CRS may be prevented by (i) providing direct protection to women and/or schoolgirls (a selective vaccination strategy); (ii) vaccinating children to provide indirect protection by reducing the transmission of rubella virus (a childhood vaccination strategy); or (iii) a combination of the two [24]. The second strategy was adopted through the 2011 SIA in Lao PDR. Although vaccinating women of childbearing age has the strongest impact as a rubella control strategy, our modelling estimates suggest that vaccinating children alone has the potential to reduce the CRS incidence.

### Stability testing

The results from the stability testing of the same vaccine that was used in the 2011 SIA indicated that the measles component of the MR vaccine is more heat sensitive than the rubella component under certain conditions. The cold chain temperature was not recorded before or during the 2011 SIA and direct evidence of heat exposure destroying measles vaccine components during the SIA does not exist. However, our field work during the survey and other approaches revealed that inadequate handling of vaccine vials and incorrect preparation of vaccines are not so rare (e.g., keeping vials out of the cold chain for several weeks or filling the vaccine into the syringe the day before injection).

Various hypotheses have been advanced to explain measles outbreaks experienced by countries that have performed SIAs with high coverage (i.e., 95%): inflow of unvaccinated immigrants, underestimated denominators due to an unreliable census, improper injection practice, weak cold chain, or a combination of these factors [25–28]. However, most of the time, these hypotheses have never been proven or disproven because no systematic analysis is conducted. In our study in Lao PDR, the reason why measles IgG prevalence increased by age (80.6% in 5–14 years old and 92.7% in 15–21 years old), but rubella did not among the target population of 2011 SIA can be partly explained by differences in heat stability between the measles and rubella vaccine components. The contribution of immunogenicity differences between measles and rubella vaccine components is not clear. Taken together, this low prevalence might cause repeated measles outbreaks in the country. Regular monitoring of immunization activities by vaccination coverage and surveillance data is essential, and more accurate estimation of population immunity than just the reported coverage (the seroprevalence survey) should be conducted when countries experience a measles outbreak after SIA with high coverage. In such countries, samples should be obtained from both SIA-targeted and -nontargeted ages from a representative population [29, 30].

### Strengths of the study

This study is the first nationwide survey of the prevalence of anti-measles IgG antibody and anti-rubella IgG antibody in a general population among SIA-targeted and -nontargeted ages. We applied multistage random cluster sampling to better represent the general population in Lao PDR. The design effect of prevalence was calculated to be between 0.8 and 2.9, which was acceptable as we set it at around 1.6 before the survey. Infants aged 1–2 years showed higher design effects (2.85 for measles and 2.62 for rubella IgG prevalence), which may reflect local epidemics and/or routine immunization coverage.

The background characteristics of our sampled population were similar to those of another nationwide population-based study, the Lao PDR Social Indicator Survey (LSIS) conducted in 2010–2011. For example, the locations of current residences—north, central, and south—were 26.9%, 26.9%, and 46.3% in our survey and 32.2%, 49.1%, and 18.7% in the LSIS. The education levels attained by mothers—none, primary school, secondary school or more—were 19.2%, 30.8%, and 50.0% in our survey and 16.4%, 32.2%, and 50.3% in the LSIS. The LSIS applied the multistage stratified cluster sampling method and surveyed more than 13,000 women all over the country. A direct comparison of the populations sampled by the two different surveys is difficult to perform as the primary objectives were different. Nonetheless, our sampled population likely represents the general population in Lao PDR.

### Limitations of the study

The effectiveness of immunization is difficult to evaluate in Lao PDR because the country has more than 30 years’ experience of routine immunization with variable annual reported coverage and several SIAs using measles-containing vaccine. Currently, no reliable laboratory method to differentiate natural exposure from vaccination is available for field epidemiological studies using dried blood spots. Therefore, the results should be interpreted carefully.

Another limitation is that the immunization records were not obtained from the SIA target age groups. Written records must provide additional valuable information on whether or not each individual was actually administered the vaccine during the 2011 SIA. However, our previous study conducted in Lao PDR in 2011 and 2012 focusing on 5–9 year olds revealed that less than one-fourth of participants had kept their records from the time of the survey [8, 9]. Thus, the collection of written immunization records from the SIA-targeted age groups is considered impractical.

There was a potential bias in our estimates of IgG prevalence among adults, given that they were sampled from parents of 1–2 year olds. Parents may likely be exposed to measles and rubella more often than adults of same age without 1–2 year old children. However, average Lao families have six children, and child adoption is very common [31], thus prevalence in parents may only be slightly overestimated.

## Conclusions

We provide a quantitative analysis of the estimated IgG prevalence among the representative population for SIA-targeted and SIA-nontargeted ages in Lao PDR. We conclude that the 2011 SIA were not sufficiently effective for measles because the estimated anti-measles IgG positivity was lower than its herd immunity threshold. The IgG prevalence increased with age despite the reported coverage of 97% in 2011, which implies that the wild measles virus is still circulating. However, the 2011 SIA appears to have been effective for rubella because of the high IgG prevalence achieved and the mathematical modelling for the rubella immunity profile revealed that a significant number of CRS cases may have been averted per year. The difference in the effectiveness of the two vaccine antigens can be in part explained by the results of the stability testing indicating that the measles component is more heat sensitive than the rubella component. This finding suggests that vaccine management was not ideal. Further study is needed to evaluate vaccine management, including vaccine temperature monitoring.

Seroprevalence surveys of measles and rubella IgG provide valuable information on population immunity and enable the effectiveness of past measles and rubella immunization activities to be evaluated. Similar seroprevalence surveys should be conducted in countries with repeated measles epidemics after SIAs which have achieved with high coverage (95%) to evaluate immunization effectiveness.

## Supporting information

S1 Table Summary of the catalytic models used in the analyses of serological data. The expressions for the age-specific proportion susceptible or seronegative for models B-D can be obtained either by setting *λ_y_* = *λ_o_* and/or setting *p* = 1.
(DOCX)

S2 Table Summary of the best-fitting estimates of the force of infection, sensitivity of the assay (where appropriate), obtained from each model. (DOCX)

S3 Table Age, sex, and anti-measles IgG and anti-rubella IgG test results. (XLSX)

## References

[1] Strebel PM, Papania MJ, Fiebelkorn AP, Halsey NA. Measles vaccine In: Plotkin SA, Orenstein WA, Offit PA eds. Vaccine 6th ed, Saunders, 2013, pp352–387.

[2] World Health Organization. Measles vaccines: WHO position paper. Wkly Epidemiol. Rec, 2009; 84: 349–360. 19714924

[3] Perry RT, Gacic-Dobo M, Dabbagh A, Mulders MN, Strebel PM, Okwo-Bele JM, et al Progress toward regional measles elimination–worldwide, 2000–2013. Morb Mortal Wkly Rep. 2014; 63: 1034–1038. PMC5779499 25393223

[4] Reef SE, Plotkin SA. Rubella vaccine In: Plotkin SA, Orenstein WA, Offit PA eds. Vaccine 6th ed, Saunders, 2013, pp688–717.

[5] World Health Organization. Progress towards eliminating rubella and congenital rubella syndrome in the western hemisphere, 2003–2008. Wkly Epidemiol Rec. 2008; 83: 393–400. 18975450

[6] World Health Organization. Rubella vaccines: WHO position paper. Wkly Epidemiol Rec. 2011; 86: 301–316. 21766537

[7] Vynnycky E, Adams EJ, Cutts FT, Reef SE, Navar AM, Simons E, et al Using seroprevalence and immunisation coverage data to estimate the global burden of congenital rubella syndrome, 1996–2010: A Systematic Review. PLoS ONE. 2016; 11: e0149160 doi: 10.1371/journal.pone.0149160 26962867 10.1371/journal.pone.0149160 PMC4786291

[8] Xeuatvongsa A, Komada K, Kitamura T, Vongphrachanh P, Pathammavong C, Phounphenghak K, et al Chronic hepatitis B prevalence among children and mothers: results from a nationwide, population-based survey in Lao People's Democratic Republic. PLoS ONE. 2014; 9: e88829 doi: 10.1371/journal.pone.0088829 24586408 10.1371/journal.pone.0088829 PMC3938412

[9] Komada K, Sugiyama M, Vongphrachanh P, Xeuatvongsa A, Khamphaphongphane B, Kitamura T, et al Seroprevalence of chronic hepatitis B, as determined from dried blood spots, among children and their mothers in central Lao People’s Democratic Republic: A multistage, stratified cluster sampling survey. Int J Infect Dis. 2015; 36: 21–26. doi: 10.1016/j.ijid.2015.04.020 25957815 10.1016/j.ijid.2015.04.020

[10] World Health Organaization. Guidance on conducting serosurveys in support of measles and rubella elimination in the WHO European Region. 2013, WHO Regional Office for Europe: Copenhagen, Denmark. http://www.euro.who.int/__data/assets/pdf_file/0011/236648/Guidance-on-conducting-serosurveys-in-support-of-measles-and-rubella-elimination-in-the-WHO-European-Region.pdf (accessed February 28, 2017)

[11] Uzicanin A, Lubega I, Nanuynja M, Mercader S, Rota P, Bellini W, et al Dried blood spots on filter paper as an alternative specimen for measles diagnostics: detection of measles immunoglobulin M antibody by a commercial enzyme immunoassay. J Infect Dis. 2011; 204 (Suppl 1): S564–9. https://doi.org/10.1093/infdis/jir088 21666214 10.1093/infdis/jir088

[12] Riddell MA, Byrnes GB, Leydon JA, Kelly HA. Dried venous blood samples for the detection and quantification of measles IgG using a commercial enzyme immunoassay. Bull World Health Organ. 2003; 81: 701–7. 14758429 PMC2572339

[13] World Health Organization. Immunological basis for immunization series. Module 7: measles update 2009. 2009.

[14] Fitter DL, Anselme R, Paluku G, Rey G, Flannery B, Tohme RA, et al Seroprevalence of measles and rubella antibodies in pregnant women Haiti, 2012. Vaccine. 2013; 32: 69–73. doi: 10.1016/j.vaccine.2013.10.071 24188751 10.1016/j.vaccine.2013.10.071

[15] Cutts FT, Vynnycky E, Modelling the incidence of congenital rubella syndrome in developing countries. Int J Epidemiol. 1999; 28: 1176–1184. 10661666 10.1093/ije/28.6.1176

[16] Mao B, Chheng K, Wannemuehler K, Vynnycky E, Buth S, Soeung SC, et al Immunity to polio, measles and rubella in women of child-bearing age and estimated congenital rubella syndrome incidence, Cambodia, 2012. Epidemiol Infect. 2015; 143: 1858–67. doi: 10.1017/S0950268814002817 25373419 10.1017/S0950268814002817 PMC9507254

[17] Alleman MM, Wannemuehler KA, Hao L, Perelygina L, Icenogle JP, Vynnycky E, et al Estimating the burden of rubella virus infection and congenital rubella syndrome through a rubella immunity assessment among pregnant women in the Democratic Republic of the Congo: Potential impact on vaccination policy. Vaccine. 2016; 34: 6502–6511. doi: 10.1016/j.vaccine.2016.10.059 27866768 10.1016/j.vaccine.2016.10.059 PMC10431197

[18] Shkedy Z, Aerts M, Molenberghs G, Beutels P, Van Damme P. Modelling age-dependent force of infection from prevalence data using fractional polynomials. Stat Med. 2006; 25: 1577–1591. doi: 10.1002/sim.2291 16252265 10.1002/sim.2291

[19] Sakata M, Komase K, Nakayama T. Histidine at position 1042 of the p150 region of a KRT live attenuated rubella vaccine strain is responsible for the temperature sensitivity. Vaccine. 2009; 27: 234–242. doi: 10.1016/j.vaccine.2008.10.049 18996422 10.1016/j.vaccine.2008.10.049

[20] World Health Organization. Immunological basis for immunization: module 11 rubella. 2008.

[21] Miyakawa M, Yoshino H, Yoshida LM, Vynnycky E, Motomura H, Tho le H, et al Seroprevalence of rubella in the cord blood of pregnant women and congenital rubella incidence in Nha Trang, Vietnam. Vaccine. 2014; 32: 1192–1198. doi: 10.1016/j.vaccine.2013.08.076 24021315 10.1016/j.vaccine.2013.08.076

[22] Toda K, Reef S, Tsuruoka M, Iijima M, Dang TH, Duong TH, et al Congenital rubella syndrome (CRS) in Vietnam 2011-2012—CRS epidemic after rubella epidemic in 2010–2011. Vaccine. 2015; 33: 3673–3677. doi: 10.1016/j.vaccine.2015.06.035 26087296 10.1016/j.vaccine.2015.06.035 PMC10792995

[23] Vynnycky E, Yoshida LM, Huyen DT, Trung ND, Toda K, Cuong NV, et al Modeling the impact of rubella vaccination in Vietnam. Hum Vaccin Immunother. 2016; 12: 150–8. doi: 10.1080/21645515.2015.1060380 26260857 10.1080/21645515.2015.1060380 PMC7002053

[24] Robertson SE, Cutts FT, Samuel R, Diaz-Ortega JL. Control of rubella and congenital rubella syndrome (CRS) in developing countries, Part 2: Vaccination against rubella. Bulletin of the World Health Organ. 1997; 75: 69–80. PMC2486979 9141752

[25] Goodson JL, Chu SY, Rota PA, Moss WJ, Featherstone DA, Vijayaraghavan M, et al Research priorities for global measles and rubella control and eradication. Vaccine. 2012; 30: 4709–4716. doi: 10.1016/j.vaccine.2012.04.058 22549089 10.1016/j.vaccine.2012.04.058 PMC10321687

[26] Cutts FT, Hanson M. Seroepidemiology: an underused tool for designing and monitoring vaccination programmes in low- and middle-income countries. Trop Med Int Health. 2016; 21: 1086–1098. doi: 10.1111/tmi.12737 27300255 10.1111/tmi.12737

[27] Plans-Rubio P. Is the current prevention strategy based on vaccination coverage and epidemiological surveillance sufficient to achieve measles and rubella elimination in Europe? Expert Rev Anti Infect Ther. 2014; 12: 723–726. doi: 10.1586/14787210.2014.917047 24807016 10.1586/14787210.2014.917047

[28] Rentsen T1, Enkhtuya B, Nymadawa P, Kobune F, Suzuki K, Yoshida H, et al Measles outbreak after a post-honeymoon period in Mongolia, 2001. Jpn J Infect Dis. 2007; 60: 198–199. 17642531

[29] Cutts FT, Lessler J, Metcalf CJ. Measles elimination: progress, challenges and implications for rubella control. Expert Rev Vaccines. 2013; 12: 917–932. doi: 10.1586/14760584.2013.814847 23984961 10.1586/14760584.2013.814847

[30] Centers for Disease Control and Prevention. Measles outbreaks and progress toward measles preelimination—African region, 2009–2010. Morb Mortal Wkly Rep. 2011; 60: 374–378. 21451448

[31] Ministry of health and Lao Statistics Buerau. Lao Social Indicator Survey 2011–2012. 2012. https://dhsprogram.com/pubs/pdf/FR268/FR268.pdf (Accessed 28 February 2017)

