# Supplementary material for: Evaluation of nationwide supplementary immunization in Lao People's Democratic Republic: Population-based seroprevalence survey of anti-measles and anti-rubella IgG in children and adults, mathematical modelling and a stability testing of the vaccine
Source: PLoS One. 2018 Mar 29;13(3):e0194931. doi: 10.1371/journal.pone.0194931 (PMC5875789; doi:10.1371/journal.pone.0194931)
Supplement: S1 Table — The expressions for the age-specific proportion susceptible or seronegative for models B-D can be obtained either by setting λy = λo and/or setting p = 1. (DOCX) [file pone.0194931.s001.docx]

**S1 Table. Summary of the catalytic models used in the analyses of serological data.** The expressions for the age-specific proportion susceptible or seronegative for models B-D can be obtained either by setting *$\lambda{}_{y}=\lambda_{o}$* and/or setting *p*=1*.*

| Model | Assumption |
| --- | --- |
| A | The force of infection differs between younger and older individuals and was estimated, and the sensitivity of the assay was unknown and was estimated, together with the force of infection. The following equations give the proportion of individuals of age *a* (*s_-_(a)*) that are seronegative and susceptible (*s_u_(a)*) for those aged over 17 years:   \|    \|  \| \| --- \| --- \|   where *p* is the sensitivity of the serological assay, *$\lambda{}_{y}$* and *$\lambda{}_{o}$* are the average force of infection among younger and older individuals before the SIA and *r* is the reduction in the force of infection as a result of the SIA. Note that only data for those aged at least 22 years were used in the fitting. |
| B | The force of infection differs between younger and older individuals and was estimated, and the sensitivity of the assay was fixed at 100%. This model is similar to that used previously[1]. |
| C | The force of infection was identical for younger and older individuals, but the sensitivity of the assay could be <100% and was identical for all ages. Both the force of infection and the sensitivity of the assay were estimated. This model is equivalent to the variable asymptote model defined by Muench[5]. |
| D | The force of infection was identical for all age groups and was estimated; the sensitivity of the assay was fixed at 100%. This model is equivalent to the simple catalytic model[5]. |
