# Supplementary material for: Evaluation of nationwide supplementary immunization in Lao People's Democratic Republic: Population-based seroprevalence survey of anti-measles and anti-rubella IgG in children and adults, mathematical modelling and a stability testing of the vaccine
Source: PLoS One. 2018 Mar 29;13(3):e0194931. doi: 10.1371/journal.pone.0194931 (PMC5875789; doi:10.1371/journal.pone.0194931)
Supplement: S2 Table — (DOCX) [file pone.0194931.s002.docx]

**S2 Table. Summary of the best-fitting estimates of the force of infection, sensitivity of the assay (where appropriate), obtained from each model.**

| Model | Assumed reduction in force of infection since 2011 | Force of infection per 1000 (susceptibles) before 2011 | | Sensitivity of the antibody assay (%) | Loglike-lihood deviance (degrees of freedom) | Weighted CRS incidence per 100,000 live births | | Number of CRS cases in 2013 | | |
| --- | --- | --- | --- | --- | --- | --- | --- | --- | --- | --- |
|  |  | <15 yr olds | ≥15 year olds |  |  | Without vaccination | With vaccination | Without vaccination | With vaccination | Prevented |
| A | 0% | 79 (41,124) | 18 (9,128) | 99 (82,100) | 61 (42) | 96 (41,453) | 86 (40,356) | 161 (69,758) | 145 (67,595) | 16 (-33,175) |
|  | 25% | 80 (45,126) | 18 (9,128) | 100 (82,100) | 61 (42) | 95 (41,434) | 64 (30,263) | 158 (69,726) | 108 (50,439) | 51 (8,292) |
|  | 50% | 82 (49,127) | 19 (9,128) | 99 (82,100) | 61 (42) | 96 (41,410) | 44 (20,175) | 160 (68,686) | 74 (33,293) | 86 (34,398) |
|  | 75% | 82 (52,129) | 19 (9,127) | 99 (82,100) | 61 (42) | 94 (41,388) | 22 (10,88) | 157 (68,648) | 36 (17,147) | 120 (52,503) |
|  | 100% | 83 (56,130) | 19 (9,127) | 99 (82,100) | 61 (42) | 93 (41,362) | - | 156 (68,605) | 0 (0,0) | 156 (68,605) |
| B | 0% | 79 (61,96) | 18 (8,29) | - | 61 (43) | 95 (37,178) | 85 (35,152) | 158 (62,297) | 142 (58,254) | 16 (0,50) |
|  | 25% | 79 (62,97) | 18 (8,29) | - | 61 (43) | 94 (37,175) | 69 (28,122) | 158 (62,293) | 116 (47,204) | 42 (11,92) |
|  | 50% | 80 (63,97) | 18 (8,30) | - | 61 (43) | 93 (37,172) | 53 (22,94) | 156 (61,288) | 89 (36,156) | 67 (21,136) |
|  | 75% | 80 (64,97) | 18 (8,29) | - | 61 (43) | 92 (36,170) | 37 (15,66) | 154 (61,285) | 62 (26,111) | 92 (32,180) |
|  | 100% | 81 (65,98) | 18 (8,29) | - | 61 (43) | 92 (36,168) | 21 (8,38) | 153 (61,280) | 35 (14,64) | 118 (43,224) |

| Model | Assumed reduction in force of infection since 2011 | Force of infection per 1000 (susceptibles) before 2011 | | Sensitivity of the antibody assay (%) | Loglike-lihood deviance (degrees of freedom) | Weighted CRS incidence per 100,000 live births | | Number of CRS cases in 2013 | | |
| --- | --- | --- | --- | --- | --- | --- | --- | --- | --- | --- |
|  |  | <15 yr olds | ≥15 year olds |  |  | Without vaccination | With vaccination | Without vaccination | With vaccination | Prevented |
| C | 0% | 76 (55,123) | 76 (55,123) | 85 (80,92) | 62 (43) | 247 (143,290) | 226 (185,260) | 414 (239,485) | 378 (309,434) | 35 (-137,100) |
|  | 25% | 78 (57,126) | 78 (57,126) | 85 (79,92) | 62 (43) | 243 (137,288) | 194 (161,231) | 407 (229,482) | 324 (269,386) | 83 (-124,164) |
|  | 50% | 80 (58,129) | 80 (58,129) | 85 (79,91) | 62 (43) | 239 (131,286) | 160 (132,206) | 399 (220,478) | 267 (221,344) | 132 (-103,230) |
|  | 75% | 82 (60,134) | 82 (60,134) | 84 (79,91) | 62 (43) | 234 (123,283) | 125 (96,189) | 391 (206,473) | 209 (161,316) | 182 (-91,300) |
|  | 100% | 84 (61,138) | 84 (61,138) | 84 (79,91) | 62 (43) | 229 (115,280) | 88 (55,166) | 383 (193,468) | 148 (91,278) | 235 (-69,371) |
| D | 0% | 42 (39,46) | 42 (39,46) | - | 78 (44) | 301 (300,301) | 238 (223,256) | 504 (502,504) | 398 (373,427) | 105 (76,131) |
|  | 25% | 43 (40,47) | 43 (40,47) | - | 79 (44) | 301 (300,301) | 194 (179,212) | 504 (501,504) | 325 (299,354) | 179 (149,204) |
|  | 50% | 44 (40,47) | 44 (40,47) | - | 80 (44) | 301 (299,301) | 148 (133,166) | 503 (500,504) | 247 (222,278) | 256 (226,281) |
|  | 75% | 44 (41,48) | 44 (41,48) | - | 81 (44) | 301 (299,301) | 100 (84,118) | 503 (500,504) | 167 (141,197) | 336 (305,362) |
|  | 100% | 45 (42,49) | 45 (42,49) | - | 82 (44) | 301 (298,301) | 49 (34,68) | 503 (498,504) | 83 (57,113) | 420 (388,446) |
